# Targeting Strategies for Renal Cell Carcinoma: From Renal Cancer Cells to Renal Cancer Stem Cells

**DOI:** 10.3389/fphar.2016.00423

**Published:** 2016-11-10

**Authors:** Zhi-xiang Yuan, Jingxin Mo, Guixian Zhao, Gang Shu, Hua-lin Fu, Wei Zhao

**Affiliations:** ^1^Department of Pharmacy, College of Veterinary Medicine, Sichuan Agricultural UniversityChengdu, China; ^2^Key Laboratory for Stem Cells and Tissue Engineering, Ministry of Education, Sun Yat-sen UniversityGuangzhou, China; ^3^Department of Histology and Embryology, Zhongshan School of Medicine, Sun Yat-sen UniversityGuangzhou, China

**Keywords:** renal cell carcinoma, renal cancer stem cells, targeting strategies, renal cancer, surface markers

## Abstract

Renal cell carcinoma (RCC) is a common form of urologic tumor that originates from the highly heterogeneous epithelium of renal tubules. Over the last decade, targeting therapies to renal cancer cells have transformed clinical care for RCC. Recently, it was proposed that renal cancer stem cells (CSCs) isolated from renal carcinomas were responsible for driving tumor growth and resistance to conventional chemotherapy and radiotherapy, according to the theory of CSCs; this has provided the rationale for therapies targeting this aggressive cell population. Precise identification of renal CSC populations and the complete cell hierarchy will accurately inform characterization of disease subtypes. This will ultimately contribute to more personalized and targeted therapies. Here, we summarize potential targeting strategies for renal cancer cells and renal CSCs, including tyrosine kinase inhibitors, mammalian target of rapamycin inhibitors (mTOR), interleukins, CSC marker inhibitors, bone morphogenetic protein-2, antibody drug conjugates, and nanomedicine. In conclusion, targeting therapies for RCC represent new directions for exploration and clinical investigation and they plant a seed of hope for advanced clinical care.

## Introduction

Renal cell carcinoma (RCC) is not a single entity, but rather comprises a population of tumors that originate from the highly heterogeneous epithelium of renal tubules. According to the Heidelberg classification of renal cell tumors, histologic subtypes of RCC include clear cell adenocarcinoma, which is the most common form of RCC, chromophobe collecting duct carcinoma, papillary carcinoma, and unclassified carcinomas (Kavacs et al., 1997). Among urologic tumors, RCC has the highest cancer-specific mortality rate, and the 5-year survival rate for patients with metastatic disease is only 12% (Siegel et al., 2015). In addition, RCC accounts for approximately 3.8% of all adult human malignancies, with an annually increasing incidence (Koul et al., 2011). Chemotherapy is one of the principal modes of cancer treatment; however, its effectiveness is limited by drug resistance. Moreover, RCC is also resistant to radiation, and weakly sensitive to immunotherapeutic agents, such as interleukin (IL)-12 and interferon (INF)-α. Tumor recurrence occurs in 40% of patients after curative surgical resection (Koul et al., 2011). To date, RCC is still a tumor of unpredictable presentation and poor clinical outcome.

Cancer stem cells (CSCs) are a small population of cancer cells responsible for resistance to radiation and chemotherapy. There are numerous mechanisms that may explain chemo-radiation resistance of CSC, including their assumed quiescence, high drug eﬄux capacity, residing in hypoxic niches, and the up-regulation of DNA damage response and repair genes (Shukrun et al., 2014). They are also responsible for metastasis, tumor development, disease progression, and disease recurrence. They are characterized by an extraordinary capacity for unlimited cell division and maintenance of the stem cell pool in the tumor. They keep dividing until they build terminally differentiated, specialized cells due to unlimited self-renewal and multipotency toward heterogeneous progeny (Visvader and Lindeman, 2008; Clevers, 2011). There is accumulating evidence that selective targeting of CSCs is possible and that CSCs can be selectively eradicated to improve the survival and quality-of-life in cancer patients (Yilmaz et al., 2006; Smith et al., 2010).

There is common consent that renal CSCs have tumorigenic ability and that they are resistant to chemo- and radio-therapy, providing the rationale for therapies targeting this aggressive cell population. The development of personalized treatment strategies is supported by comprehensive molecular analyses of RCC (Gerlinger et al., 2012; Bielecka et al., 2014) and pharmacogenomics studies of putative CSCs (Crea et al., 2011). In terms of selective markers for CSCs in clear cell RCC (ccRCC), CD105, a prominent marker, can distinguish a rare subpopulation of cells that exhibit CSC properties (Bussolati et al., 2008, 2011). CD133, also known as prominin-1, is a transmembrane glycoprotein that has been investigated as another putative marker of renal progenitor cells in the adult human kidney; it appears to be non-tumorigenic and it is expressed in resident adult kidney progenitors cells (Bruno et al., 2006). The side population (SP), which can be identified using a Hoechst 33342 dye eﬄux assay, can also express putative CSC markers, but it has not been reliably demonstrated that SP also displays CSC properties (Addla et al., 2008).

Specific CSC markers could represent valuable tools for targeting these cells with drugs or immunotoxins. Investigation of intracellular pathways and genetic abnormalities may lead to the development of efficient therapeutic strategies targeting renal CSCs (Bussolati et al., 2013). The development of a slew of rationally designed targeted therapies that inhibit vascular endothelial growth factor (VEGF) receptor and mammalian target of rapamycin (mTOR) pathways has contributed to the systemic management of RCC. Furthermore, molecular targeting nanomedicine-based approaches, which can control drug delivery and release more efficiently, can effectively inhibit multiple types of CSCs. In this review, the author summarizes the potential targeting strategies underlying tyrosine kinase inhibitors, mTOR inhibitors, interleukins, CSC marker inhibitors, bone morphogenetic protein-2 (BMP-2), antibody drug conjugates (ADCs), and nanomedicine that can target renal cancer cells and renal CSCs to treat RCC more efficiently.

## Normal Renal Stem Cells

According to the “hierarchical” theory, the three most common RCC histologic subtypes (undifferentiated, clear-cell, papillary carcinomas) may arise from the stem cell pool of the adult kidney or from the deriving progenitors. Whereas the Wilm’s tumor may arise from the embryonic renal stem cell compartment (Bussolati et al., 2011). To isolate and characterize of renal stem cells, several methods have been attempted. These include pulse-chase labeling of slow cycling cells, dye exclusion assay, FACS/magnetic bead separation by stem cell surface markers, and selective culture conditions for renal dissociated tissue (Gupta et al., 2006; Sagrinati et al., 2006; Gupta and Rosenberg, 2008; Ronconi et al., 2009). The isolated cells exhibited multipotent differentiation capacity *in vitro*. Importantly, they can integrate into injured renal tubules. The parietal epithelium of Bowman’s capsule, which give rise to both podocytes and tubules, has been suggested harbor intrarenal stem cells. In addition, multipotent CD133^+^/CD24^+^ stem cells have been further isolated from the Bowman’s capsule. CD133^+^ renal progenitor cells have been characterized in the renal cortex from the tubule/interstitium (Sagrinati et al., 2006; Lindgren et al., 2011). Humphreys et al. (2008) evaluated the degree of involvement by extratubular cells during renal regeneration. Their results demonstrated that the proliferating intratubular epithelium accounts for tubular regeneration (Humphreys et al., 2008). However, the putative connection between renal stem cells and renal carcinomas is still not clarified due to the unequivocal understandings of the renal stem cell. For example, numerous technical limitations hindered the efforts for the isolation and characterization of renal stem cells; and there are different stem cell subpopulations with restricted differentiation potential in kidneys.

## Renal Cancer Stem Cell Markers

Multiple studies have been designed to isolate and characterize the CSC population in RCC using stem cell markers, which are considered targets in CSC therapy. A better understanding of stem cell markers and the related signaling pathways that contribute to tumor progression and metastasis is important for the development of strategies for CSC-targeting treatments.

## Stem Cell Surface Markers in Renal CSCs

CD105, also called endoglin, is a receptor for transforming growth factor-β (TGF-β) and is expressed on the cell surface. It regulates cellular proliferation, differentiation, and migration, and is therefore important for angiogenesis (Dallas et al., 2008). By magnetically sorting cells in RCC specimens, Bussolati et al. (2008) separated a subpopulation of cells expressing the mesenchymal marker CD105. This finding is consistent with the mesenchymal origin of the kidney and the mesenchymal phenotype of stem cells found in normal rodent kidney as well as in the human embryo; these cells represented less than 10% of the total tumor mass (Bussolati et al., 2008). They could differentiate into epithelial cells both *in vitro* and *in vivo*. *In vivo*, CD105-positive (CD105^+^) cells could display the tumor-initiating activity that as few as 100 cells could generate transplantable tumors. *In vitro* they could differentiate into both epithelial and endothelial cells. These results indicated that the CD105^+^ cells may originate from resident renal stem cells with mesenchymal characteristics. In addition, other studies have reported that CD105^+^ cells are present in the established RCC cell lines 786-O, 769-P, ACHN, Caki-1, Caki-2, SMKTR2, SMKTR3, and RCC-6 (Khan et al., 2012, 2013) and that there is no relationship between CD105 and gender, age, cell type, or tumor size (Sandlund et al., 2006). More recently, a CSC differentiation strategy test was conducted on CD105^+^ CSCs to aid in their isolation from human renal carcinomas. CD105^+^ CSCs differentiated into cells that expressed epithelial markers (E-cadherin and pan-cytokeratin) when they were treated with recombinant human IL-15 (rhIL-15) at a concentration of 10 pg/mL. Compared with severe combined immunodeficiency (SCID) mice that were injected with untreated CD105^+^ CSCs, SCID mice with IL-15-treated CSCs demonstrated significantly higher levels of apoptosis in differentiated epithelial cells following treatment with vinblastine or paclitaxel (Azzi et al., 2011). In patients, high CD105 levels are associated with higher tumor stage and CD105 is a crucial indicator of clinical outcome. Therefore, further investigation of potential therapeutic targets is warranted.

CD133, a five transmembrane domain glycoprotein, belongs to the prominin family. It contains two large extracellular and two small intracellular loops (Grosse-Gehling et al., 2013). Currently, it serves as a useful marker for the isolation and characterization of various types of stem and progenitor cells in human tissues. Using a specific monoclonal antibody, human CD133 was first isolated from hematopoietic stem cells (HSCs) which consisted of various kinds of stem/progenitor cells and differentiated cells. The CD133^+^ cell population can influence tumor vascularization and angiogenesis, and it is also expressed in normal adult human kidney cells (Bussolati et al., 2005). Zhang et al. (2013) observed that CD133 expression was associated with stage, histological type, tumor location, and tumor grade. Bruno et al. (2006) demonstrated that CD133^+^ progenitor cells derived from human RCC contributed to tumor vascularization. High expression of CD133 is associated with a macro-/micro-cystic pattern, non-metastatic disease, and non-sarcomatoid changes (Kim et al., 2011). CD133 may have a role in risk stratification; its overexpression was associated with longer survival in patients with ccRCC. On the other hand, low CD133 expression is an independent predictor of poor disease-specific survival (DSS) and progression-free survival (PFS) (Costa et al., 2012). Additionally, CD133 may be involved in both epithelial and endothelial differentiation *in vitro* and *in vivo*. However, CD133^+^ cells were not able to induce carcinoma development after subcutaneous injection into SCID mice, indicating non-tumorigenic potential. This result is in contrast with the idea that renal CSCs arise from renal progenitors expressing the CD133 marker (Bussolati et al., 2011). Interestingly, injection of CD133^+^ cells with the RCC cell line K1 into SCID mice would lead to significant tumor growth and progression. Inside the tumor, the newly formed vessels expressed both human CD31 and human HLA class I. The resultant tumor vessels via the differentiation of CD133^+^ progenitor cells plus K1 cells was later confirmed by fluorescence *in situ* hybridization for human origin of chromosome X (Bruno et al., 2006). However, clinical significance of CD133 expression in human RCC is inconsistent. In addition, no study showed that CD133 can serve as a therapeutic target for renal cancer or renal CSCs because of its wide expression in kidney progenitor cells probably. But many papers have reported targeting CD133^+^ cells therapy for other cancers. More recently, Qi et al. (2016) loaded chemotherapeutic antitumor drugs and small interfering RNA (siRNA) against CD133^+^ into mesoporous silica nanoparticles (MSNPs) with thermo/pH-coupling sensitivity and site-specificity. These MSNPs successfully inhibited its growth *in vivo* in a laryngeal cancer mouse model by eliminating CD133^+^ cells (Qi et al., 2016).

CD44, a single-chain transmembrane glycoprotein, binds primarily to the extracellular glycosaminoglycan hyaluronan, an adhesion molecule for the extracellular matrix (Ponta et al., 2003; Hiraga et al., 2013). This interaction is considered a signaling platform for integrating cellular microenvironmental cues with growth factor and cytokine signals. Moreover, Debeb et al. (2010) described CD44^+^/CD24^-^ cells with several CSC features in the human embryonic cell line 293T. Although CD44^+^ human carcinomas are very resistant to therapy and highly malignant, there is still some debate on the role of CD44 in CSCs. Undoubtedly, metastatic potential is improved by the expression of CD44, and metastatic properties are often linked to CSCs (Toole, 2009; Zhang et al., 2013). Furthermore, CD44^+^/CD24^-^ cells in three-dimensional (3D) spheres were reported to be resistant to radio- and chemo-therapy (Debeb et al., 2010). In another report, Lim et al. (2008) suggested that CD44 expression in RCC could be used to inform the appropriate therapy and provide useful prognostic information in both primary and metastatic RCC. Meanwhile, a meta-analysis of the literature showed that CD44 was a poor prognostic marker for 5-year overall survival and that high CD44 expression levels were associated with high Fuhrman grade and recurrence (Li et al., 2015). A new study proposed by Ma et al. (2016) proposed a possible mechanism for CD44 upregulation. They found that a third of the tumor samples analyzed expressed CD44, and that while CD44 expression was not associated with clinical outcome, it was associated with a high density of tumor-associated macrophages (Ma et al., 2016).

CXCR4 is a 352-amino acid rhodopsin-like G-protein coupled receptor (GPCR) which binds selectively to the CXC chemokine stromal cell-derived factor-1 (SDF-1, also known as CXCL12). CXCR4 is expressed on all kinds of tissues and organs and normal stem cells. Therefore, it is one of the best researched chemokine receptors (Weitzenfeld and Ben-Baruch, 2014). The expression of CXCR4 has a significant negative predictive value on PFS in patients treated with IFN-α (Li et al., 2011). Low or absent CXCR4 expression is predictive of a good response to sunitinib therapy, while CXCR4 expression is associated with a poor response to sunitinib in metastatic ccRCC (D’ Alterio et al., 2012). Furthermore, the CXCL12-CXCR4 signaling axis plays an important role in the homing of normal stem cells (Lapidot and Kollet, 2002). This CXCL12-CXCR4 axis is involved in the trafficking/metastasis of CSCs to various organs when CXCR4 is expressed; CXCR4 is also highly expressed in the lymph nodes, lungs, liver, and bone (Kucia et al., 2005; Mukherjee and Zhao, 2013). Gassenmaier et al. (2013) compared the capacity of two RCC cell lines, RCC-26 and RCC-53, to form spheres *in vitro* and to establish tumors *in vivo*, and observed that CXCR4 was present only in the more tumorigenic RCC-53 cell line (Gassenmaier et al., 2013). Micucci et al. (2015) recently reported that the self-renewing ability of CXCR4^+^ spheres was enhanced before the upregulation of hypoxia-inducible factor 2α (HIF-2α) in the RCC cell lines Caki-1, Caki-2, 786-O, and 769-P. Knockdown of HIF-2α abolished CXCR4^+^ sphere formation and expression, and inhibition of HIF-2α abrogated tumor growth *in vivo*, proving that HIF-2α activation plays an important role in CSC expansion.

## Intracellular Markers in Renal CSCs

Cancer stem cells are likely derived from differentiated cancer cells through various mechanisms, including cellular interactions, tumor niche signals, and epithelial to mesenchymal transition (EMT) (Schieber and Chandel, 2013; Pattabiraman and Weinberg, 2014). EMT is a highly conserved cell process involved in normal embryogenesis and tissue repair (Liu and Fan, 2015). Recently, numerous studies have shown that EMT plays a momentous role in tumor progression. Through EMT, tumor cells can obtain stem cell-like characteristics, through the acquisition of mesenchymal properties. The results of accumulating studies have indicated that a promising link exists between EMT and the CSC phenotype. Lichner et al. (2015) reported that isolated RCC spheres displayed CSC-like properties that were linked to the TGF-β-EMT axis. Additionally, recent research has suggested that EMT can be artificially induced during the culture of the RCC cell lines ACHN and 786-O, enriching their stem cell-like characteristics (Liu and Fan, 2015). ACHN and 786-O cells treated with 50 ng/ml tumor necrosis factor-α lost their epithelial morphology and acquired a mesenchymal appearance with increased expression of mesenchymal protein markers such as vimentin, slug, and ZEB1.

Li et al. (2014) proposed a method to reverse the EMT process and inhibit CSC-like features in RCC. According to their results, honokiol extract isolated from magnolia bark – a multifunctional antiangiogenic and natural antitumor agent – can simultaneously abrogate proliferation, reverse EMT (as determined by an increase in epithelial markers and a decrease in mesenchymal markers), and significantly inhibit the CSC properties of RCC cells *in vivo* (tumorigenicity) and *in vitro* (sphere formation, invasion/migration, SP) (Fried and Arbiser, 2009; Li et al., 2014; Weygant et al., 2015). Of note, a recent study found that high expression of the PIK3R1 protein was associated with RCC progression and metastasis (Lin et al., 2015). A PIK3R1 knockout functional study demonstrated that PIK3R1 plays a crucial role in RCC cell proliferation and migration; maintenance of a mesenchymal morphology; and the incremental expression of EMT-related factors *in vitro* (Lin et al., 2015). By analyzing data in The Cancer Genome Atlas’ Kidney Renal Clear Cell Carcinoma (TCGA KIRC) dataset, Weygant et al. (2015) demonstrated that doublecortin-like kinase 1 (DCLK1) regulates EMT and maintains stemness features; DCLK1 is overexpressed in RCC tumors regardless of disease stage. In primary RCC-Caki-2 cells, silencing of the DCLK1 gene using DCLK1 siRNA resulted not only in decreased expression of EMT transcription factors (SNAI1, SNAI2, TWIST1, ZEB1, and Vimentin), but also reduced expression of the pluripotency and stemness factors MYC, Nanog, Oct-4, Sox-2, and aldehyde dehydrogenase (ALDH) 1A1 (Weygant et al., 2015). These results corroborated the finding that DCLK1 knockdown can inhibit the invasive and metastatic ability of RCC cells.

Recently, SP cells from an RCC cell line were genetically modified to knock out DnaJ (Hsp40) homolog, subfamily B, member 8 (DNAJB8) and examine its function in the tumorigenicity of RCC (Yamashita et al., 2016). Compared with non-SP RCC cells, the SP is distinguishable by increased sphere-forming ability *in vitro*, enhanced proliferative potential, and greater expression levels of β-catenin, Sonic Hedgehog, Notch, and Pax-2 stem cell markers. Furthermore, the SP had an abundance of ALDH-positive cells, suggesting that ALDH enzymatic activity may be associated with the SP (Ueda et al., 2013). DNAJB8 knockout cells displayed a decreased ratio of SP to non-SP cells and reduced sphere-forming ability, indicating that DNAJB8 contributes to CSC-like phenotype of RCC (Nishizawa et al., 2012). This study also demonstrated that the overexpression of DNAJB8 increased the proportion of SP cells, boosted tumor-initiating ability, and enhanced the expression of stem cell markers (Nishizawa et al., 2012). Furthermore, western blots and immunostains of SP cells have shown that these cells express DNAJB8 protein, indicating stem cell-like phenotypes. In DNA vaccination experiments, DNAJB8, as a potential marker of RCC CSCs, was considered as an immunotherapeutic target.

MicroRNAs (miRNAs) have been described in various cancers, including prostate cancer, glioblastoma, and breast cancer (Liu and Tang, 2011; Shimono et al., 2015). They are non-coding, small, single-stranded RNA molecules, that play a significant role in posttranscriptional gene regulation and are essential in sustaining normal pluripotent embryonic stem cells in mice (Melton et al., 2010). Analysis of the effect of miR-17 on RCC spheres with cancer stem-like properties revealed that spheres from ACHN and Caki-1 cell lines demonstrated greater tumorigenic capacity and elevated expression of mesenchymal markers and stem cell *in vivo* (Lichner et al., 2015). miR-17 overexpression hindered sphere formation by modulating the TGF-β-EMT axis (Petrocca et al., 2008). Interestingly, miR-17 inhibition led to increased colony formation in ACHN and Caki-1 cell lines, which demonstrated that miR-17 could regulate the self-renewing properties of the cells; inhibition also enhanced expression of mesenchymal markers and cancer stem markers (Giron-Michel et al., 2012). In summary, miR-17 belongs to a carcinogenic miRNA cluster necessary for development and homeostasis (Concepcion et al., 2012). Further research on miRNAs in RCC tumor promotion, therapy resistance, metastasis, progression and relapse will contribute to a better understanding of RCC tumor biology (Liu and Tang, 2011).

## Potential Targeting Strategies

**FIGURE 1:**
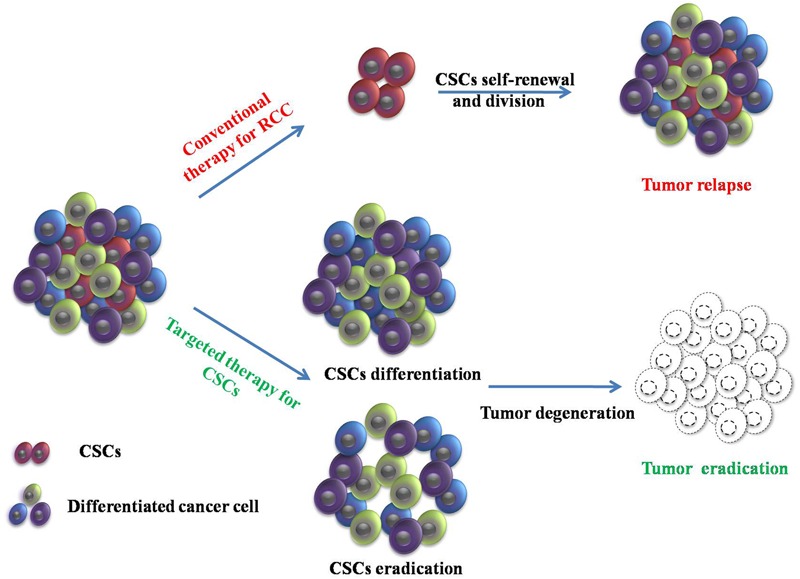
**Conventional therapy and targeting therapy for cancer stem cells (CSCs) in renal cell carcinoma (RCC).** Conventional therapy in RCC can reduce tumor size. However, CSCs are resistant to conventional therapies and induce tumor relapse through self-renewal and division. Targeted therapy for CSCs provides a new direction to eradicate the tumor.

In the view of the resistance to chemo- and radio-therapies (Lyakhovich and Lleonart, 2016) and the weak response to immunotherapeutic agents (Di Lorenzo et al., 2010) demonstrated by RCC, more attention has been paid to the development of potential approaches to target renal CSCs. Over the past several years, more and more encouraging data have demonstrated proof of principle that targeting CSCs could block the pathways associated with drug resistance, disease progression, aggressiveness, and metastasis, and may lead to complete and durable regression; this could improve treatment outcomes for patients with RCC and affect prognosis (**Figure 1**) (Sehl et al., 2009; Smith et al., 2010; Azzi et al., 2011). In particular, strategies that can target markers expressed on the cell surface or disrupt specific signaling pathways are considered ideal targeting strategies for CSCs. Identification of potential targets in the renal CSC microenvironment or nanocarriers for delivery of active compounds may also result in the development of efficient strategies for the elimination of CSCs. Potential targeting strategies for renal cancer cell and renal CSCs are summarized in the next section.

## Tyrosine Kinase Inhibitors

Tyrosine kinase signaling is a unique biochemical mechanism utilized by intra- and inter-cellular communication pathways in metazoans. Abnormal tyrosine kinase signaling leads to various cancers, as well as other diseases (Levitzki, 2013). Tyrosine kinase inhibitors (TKIs) are novel therapies that target specific cellular signaling pathways by primarily blocking tyrosine kinase receptors that are involved in the progression of several tumors, including RCC (Eichelberg et al., 2008; Albiges et al., 2012). By inhibiting the activity of growth factor receptors, in particular, VEGF receptor, platelet-derived growth factor (PDGF) receptor, and the stem cell factor c-KIT receptor, TKIs can significantly affect tumor angiogenesis and/or tumor cell proliferation. Sunitinib and sorafenib, commercially available TKIs, are associated with good treatment response and are tolerable in RCC patients. However, severe side effects including cardiotoxicity, hypothyroidism, hypertension, fatigue, hair depigmentation, hand-foot syndrome, and gastrointestinal perforation have been observed (Cohen and Oudard, 2012; Sternberg et al., 2015; Jang et al., 2016). Importantly, Czarnecka et al. (2015b) recently reported that a much more severe side effect, chronic myeloid leukemia (CML), developed in some patients with ccRCC during TKI treatment. The authors described a potential molecular mechanism of action of TKIs on bone marrow cells that may be responsible, in part, for development of CML (Czarnecka et al., 2015b).

Several pathways involved in oncogenesis are blocked by TKIs, including pathways in growth, survival, proliferation, and, most importantly, differentiation and apoptosis. However, TKIs have not been considered a primary therapy for CSCs because they lack sufficient selectivity for this peculiar cell type. Poor selectivity is also the main reason for many of the side effects of TKIs. Recent studies have demonstrated that TKIs can decrease the proliferation rate of renal CSCs, but that the efficacy of the growth inhibition was limited by hypoxic conditions and 3D intratumoral cell–cell interactions (Czarnecka et al., 2016). More evidence is needed to prove that TKIs have an inhibitory effect on renal CSCs and to delineate their mechanism of action in this cell type. Thus, a better understanding of this effect will lead to more successful therapies for RCC.

## mTOR Inhibitors

Mammalian target of rapamycin inhibitors, an important therapeutic target for RCC, permits protein translation that drives cell growth, proliferation, and the production of angiogenic growth factors including HIF-1, VEGF, and PDGF. In RCC, mTOR is stimulated by a VEGF-induced phosphorylation cascade, which involves PI3K and Akt (Czarnecka et al., 2015a; Xia and Xu, 2015). Activation of the mTOR pathway leads to translation of proteins involved in cell cycle progression and HIF-1α expression, which is associated with RCC pathogenesis. On the other hand, mTOR inhibitors whose mechanism of action is distinct from that of the TKIs mentioned above, halt tumor cell growth and angiogenesis by down-regulating HIF expression and blocking proliferation and survival signals downstream of the VEGF receptor (Kornakiewicz et al., 2014; Liu et al., 2016). Temsirolimus and everolimus are orally administered mTOR inhibitors used for targeted therapy in metastatic renal cell carcinoma (mRCC). Clinical trials in patients with mRCC showed that treatment with temsirolimus or everolimus prolonged PFS relative to the placebo in patients with mRCC that had progressed on other targeted therapies (Motzer et al., 2008; Negrier, 2008). To date, it is unknown if mTOR inhibitors may prove to be selective enough for renal CSCs and research on their selectivity is ongoing. Interestingly, some clues can be found from a number of recent studies on mTOR inhibitors that eradicated the CSC populations in nasopharyngeal carcinoma, colon cancer, and glioblastoma multiforme, among other cancers (Francipane and Lagasse, 2013; Sami and Karsy, 2013; Kolev et al., 2015; Yang et al., 2015), which may indicate future directions for designing novel drugs targeting mTOR for renal CSCs.

The emerging perspective for mTOR inhibitors in RCC treatment involves combination therapies targeting both renal cancer cells and renal CSCs. This approach requires analysis of multiple overlapping signaling networks to carefully preselect suitable molecular targets on these cells (Czarnecka et al., 2015a). However, the researchers are facing a greater challenge for trying to find new molecular targets since current knowledge on the systems biology of renal cancer cells and renal CSCs are far from enough. Therefore, attempts have been made to develop a combination treatment consisting of mTOR inhibitors and TKIs. A combination of everolimus and sorafenib was used in the treatment of mRCC; this combination had acceptable toxicity levels and demonstrated antitumor activity in previously untreated patients with mRCC (Amato et al., 2012). Another combination consisting of temsirolimus plus bevacizumab, an mTOR inhibitor, and a VEGF inhibitor, showed encouraging evidence in a Phase 1 trial for the treatment of mRCC. However, the results of the Phase 2/3 trial did not show a sufficient improvement in efficiency (Rini et al., 2014; Jobard et al., 2015). Additional combinations using mTOR inhibitors together with other targeted agents can more effectively inhibit mTOR and/or block signaling in multiple pathways, such as the PI3K/Akt/mTOR and Ras/Raf/MEK/Erk pathways (Asati et al., 2016; McKay et al., 2016).

Indeed, it remains to be seen if novel combination therapies using mTOR inhibitors can target both renal cancer cells and renal CSCs. Several studies have successfully used combination treatments to target CSCs in other cancers. A combination treatment in glioblastoma multiforme uses differentiating agents with mTOR pathway inhibitors to target CSCs, thereby targeting the ERK1/2 pathway. This underscores the importance of including differentiating agents along with inhibitors of mTOR pathways in the treatment of glioblastoma multiforme (Friedman et al., 2013). Similar studies confirmed that mTOR inhibitors combined with other agents represented promising therapeutic strategies to eradicate CSCs in breast cancer, liver cancer, and pancreatic cancer (Zhu et al., 2012; Gedaly et al., 2013; Sharma et al., 2015).

Beyond temsirolimus and everolimus, there have been a slew of second generation mTOR inhibitors, such as mTORC_1_/mTORC_2_ inhibitors and mTOR/PI3K dual inhibitors, that have shown promising activity in pre-clinical studies or have entered clinical trials (Vilar et al., 2011; Zhang et al., 2011). According to the NIH database^1^, over 50 clinical trials, ongoing or completed, proposed to apply mTOR inhibitors for the treatment of RCC. Regardless of the potential effect of novel mTOR inhibitors on CSCs, it may be necessary to combine them with other drugs to ensure an antiangiogenic effect. Combinatorial control of cell signaling still seems to be more successful than monotherapy.

## Interleukins

The interleukins act as ligands and regulate many physiologic and pathophysiologic processes by binding to their receptors and activating or inhibiting several intracellular pathways. This has led to the investigation of these pathways as potential therapeutic targets. Interleukins-1, -2,-3, -4, -6, -11, -12, -15, -18, and -21 have been tested in the clinic with variable rates of success and failure as cancer therapeutics (Aulitzky et al., 1994; Dranoff, 2004). IL-2-based immunotherapy has been approved by the FDA and is the standard therapy for advanced RCC, even though poor response and severe toxicity can occur in some cases (Di Lorenzo et al., 2010). The response rate with IL-2 treatment ranges from 7 to 27%. The best response was achieved in patients with favorable Motzer criteria, ccRCC, and lung metastases only. Long-term complete responders have been reported with high-dose bolus IL-2 regimens in ccRCC (Ljungberg et al., 2010).

**FIGURE 2:**
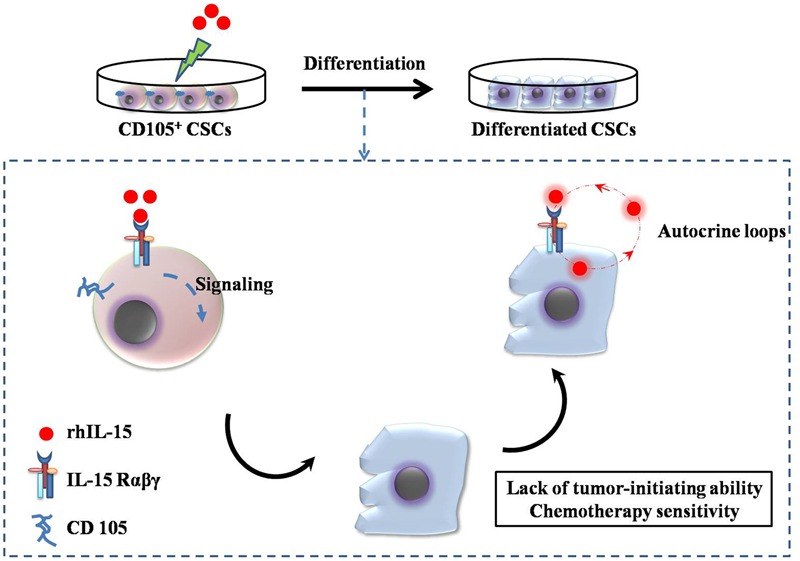
**Interleukin (IL)-15-based targeting strategy for renal CSCs.** CD105^+^ CSCs identified and isolated from human renal carcinomas were cultured with 10 pg/mL recombinant human IL-15, which induced the loss of stem cell markers and induced epithelial differentiation via the signaling pathway (IL-15Rαβγ chains). The differentiated CD105^+^ CSCs lost their tumor-initiating and self-renewing abilities. Endogenous IL-15-based autocrine loops were activated to induce permanent epithelial differentiation.

IL-15 exhibits a therapeutic index superior to that of IL-2, and is commonly used in renal cancer treatment (Steel et al., 2012; Waldmann, 2014). Although some of the biological properties of IL-15 overlap with those of IL-2, such as the activation of natural killer (NK) cells and CD8 T cells, IL-15 is not associated with capillary leak syndrome or activation-induced cell death, nor does it have a major effect on the number of functional regulatory T cells (Waldmann et al., 2011). Systemic administration of rhIL-15 showed less toxic effects than IL-2, indicating that multiple daily infusions of rhIL-15 could be administered to patients with metastatic malignancies (Munger et al., 1995; Waldmann et al., 2011). Recently, IL-15 was shown to induce differentiation of CD105^+^ renal CSCs isolated from human renal carcinomas (Azzi et al., 2011). Based on previous studies that had shown that IL-15 acted as a survival factor in renal tubular epithelial cells via autocrine-paracrine loops, the authors identified IL-15 as a potential candidate (Shinozaki et al., 2002). Other studies have shown that IL-15 upregulates E-cadherin expression through γc chain signaling pathway and blocks EMT in renal tubular epithelial cells (**Figure 2**) (Giron-Michel et al., 2012, 2013). Azzi et al. (2011) demonstrated that IL-15 can induce the stable epithelial differentiation of renal CD105^+^ CSCs, thereby generating a differentiated non-tumorigenic cell population that is more sensitive to the chemotherapeutic drugs vinblastine and paclitaxel, compared with the parental CSCs. RCC cells do not secrete IL-15, suggesting that the loss of intratumoral IL-15 secretion could be a protection mechanism for CSC stemness (Trinder et al., 1999; Azzi et al., 2011). Finally, self-renewal along with tumor sphere-forming ability was blocked in renal CSCs treated with IL-15, indicating the potential utility of IL-15 as a differentiation therapy in RCC. These observations support that IL-15 is a key factor directing the epithelial differentiation of renal CSCs. Moreover IL-15 therapy meets the criteria of CSC targeting treatment strategy: depletion of CSCs pool and generation of differentiated non-tumorigenic cells with increased sensitivity to chemotherapeutic drugs. Azzi et al. (2011) proposed two IL-15-based therapy strategies to target renal CSCs: (1) induction of paracrine IL-15 secretion by renal CSCs or neighboring cells mediated by gene transduction; (2) direct administration of IL-15 to the tumor tissue or delivery to the CSCs by coupling to peptides or antibodies which target to renal cancer cells or renal CSCs markers. Due to the potential dual effect of IL-15 on both the differentiation of renal CSCs and the activation of an antitumor immune response, this therapy strategy could provide the foundations for the enhancement of renal carcinoma treatment. A phase 1 study of the effect of intravenous human rhIL-15 in the treatment of mRCC has been completed recently (NCT01021059)^2^.

IL-21, a novel cytokine with structural and sequence homology to IL-2 and IL-15, has been studied extensively both *in vitro* and *in vivo* and evaluated in Phase 1 and 2 trials (NCT00617253, NCT00389285, and NCT00095108) in mRCC. The data from these trials indicated that IL-21 has an acceptable safety profile and encouraging single agent activity in early phase RCC and melanoma clinical trials (Hashmi and Van Veldhuizen, 2010). To date, it has not been shown if IL-21 has utility as a targeted therapy, similar to IL-15, in renal CSCs. Further investigation is needed to determine the effect of IL-21 on renal cancer cells or CSCs in the treatment of RCC.

## CSC Surface Marker Inhibitors

### CD105/Endoglin Inhibitors

Renal CSCs isolated from nephrectomy specimens express CD105/endoglin, allowing valuable therapeutic target for the tumor vasculature (Bussolati et al., 2008). The identification of CD105/endoglin as a specific CSC marker was a solid buildup for treatment of RCC and its biology significance has been discussed as above. CD105 expression is also upregulated by hypoxia through induction of HIF-1α or following inhibition of the VEGF pathway (Seon et al., 2011). It is appealing to consider that the identification and inhibition of CSC markers may result in greater treatment efficacy in RCC. The evaluation of anti-CD105/endoglin antibodies in RCC is a logical next step.

TRC105, a novel chimeric IgG1 anti-CD105/endoglin monoclonal antibody with high avidity, induces antibody-dependent cellular cytotoxicity and apoptosis in human umbilical vein endothelial cells and CD105^+^ tumor cells (Rosen et al., 2012). Notably, the angiogenesis inhibition of TRC105 was much more impressive than the inhibition of human endothelial proliferation *in vitro*, emphasizing the importance of assays that mimic the interplay between endothelium and perivascular cells during angiogenesis (Rosen et al., 2014). TRC105 is currently being evaluated for the treatment of RCC in Phase 1 and 2 trials (NCT01727089 and NCT01806064)^2^ (Choueiri et al., 2015a; Dorff et al., 2016). Encouraging preliminary results showed that administration of 10 mg/kg TRC105 was well tolerated with axitinib in patients with RCC. In addition, no TRC105-associated dose-limiting toxicities were observed with doses ranging from 8 to 10 mg/kg. Most importantly, the combination of TRC105 and axitinib increased the overall response rate and doubled PFS compared with axitinib as a single agent (Choueiri et al., 2015b).

### CXCR4 Inhibitors

The CXCL12/CXCR4 biological axis, also discussed above, is another potential therapeutic target for RCC. It is believed to play significant roles in tumorigenesis and metastasis (Gassenmaier et al., 2013). Small-molecule antagonists and other strategies to downregulate CXCR4 expression have already been developed; these represent potential therapeutic strategies to inhibit tumors metastasis and enhance the survival of patients with RCC. AMD3100 (commonly known as plerixafor, trade name Mozobil) is a specific CXCR4 antagonist that inhibits cancer cell tumorigenesis, disrupts the CSC niche, and sensitizes CSCs to chemotherapy (Yang and Wechsler-Reya, 2007). Distinct from the commonly used method for the design of antagonists, three cyclic peptides were designed by Portella et al. (2013) as new CXCR4 antagonists. These three CXCR4 inhibitory peptides were proven to be effective in inhibiting CXCR4-dependent migration and binding, P-ERK1/2-induction, calcium eﬄux, and tumor growth of renal cancer cells (Portella et al., 2013). LY2510924 is a peptide antagonist of CXCR4 from Eli Lilly and Company and is currently in Phases 1 and 2 testing to evaluate its safety and efficacy in RCC (NCT01391130)^3^. Preliminary data suggested that adding the CXCR4 inhibitor LY2510924 to sunitinib as a first-line treatment for metastatic RCC was tolerated but did not improve efficacy (Hainsworth et al., 2015, 2016).

RNA interference (RNAi), which has been proven to be a powerful tool for suppressing CXCR4 gene expression, has also been used as a treatment strategy in RCC. Wang et al. (2012b) developed recombinant CXCR4-RNAi plasmids that they transfected into RCC A-498 cells overexpressing CXCR4 *in vitro*. Specific downregulation of CXCR4 by RNAi inhibited cell growth, invasion, and migration and induced cell apoptosis in RCC *in vitro* (Wang et al., 2012b).

Ierano et al. (2014) demonstrated that mTOR activation was specifically inhibited by CXCR4 antagonists in human renal cancer cells. CXCR4 antagonists seem to regulate mTOR signaling in renal cancer cells, offering new therapeutic opportunities and targets to overcome resistance to mTOR inhibitors (Ierano et al., 2014). Similarly, a newly discovered CXCL12 receptor, CXCR7, is highly expressed in RCC patients, indicating that CXCR7 may be useful a novel surface marker and therapeutic target for RCC (Maishi et al., 2012; Wang et al., 2012a).

## Bone Morphogenetic Protein-2

Bone morphogenetic protein-2 is a member of the TGF superfamily. It regulates various cellular processes including cell differentiation, proliferation, morphogenesis, cellular survival, and apoptosis, and has been shown to have an inhibitory effect in many tumor types (Ye et al., 2013; Zhang et al., 2014; Zheng et al., 2014). However, the inhibitory effect of BMP-2 in tumors is controversial and some studies have shown that it stimulates tumor growth (Carragee et al., 2013; Liao et al., 2015), indicating that the action of BMP-2 on tumor growth probably depends on the cancer type. Most recently, it has been reported that BMP-2 inhibits tumor growth in human RCC and induces bone formation (Wang et al., 2012c). This study revealed that BMP-2 inhibited growth of the ACHN and Caki-2 cell lines, human RCC cell lines that express three types of BMP receptors. This anti-proliferative effect seems to be due to cell cycle arrest in the G1 phase. A follow-up study demonstrated that BMP-2 could inhibit the tumor-initiating ability of human renal ALDH^+^ CSCs, downregulate expression of embryonic stem cell markers, and upregulate transcription of osteogenic markers, suggesting that BMP-2 may therefore represent a beneficial treatment strategy for human RCC by targeting the CSC-enriched population (Wang et al., 2015). Mitsui et al. (2015) revealed that impaired regulation of BMP-2 via epigenetic pathways was associated with RCC pathogenesis, supporting a role for BMP-2 as a therapeutic strategy in RCC. However, the poor absorption and instability of BMP-2 means that further research is needed to improve its drugability by pharmaceutical technologies; this could include PEGylation or packaging into liposomes or nanoparticles.

## Antibody Drug Conjugates (ADCs)

An ADC is comprised of a monoclonal antibody conjugated to a cytotoxin that specifically binds to cell-surface antigens on the target cell; it represents a novel method for the treatment of mRCC (Goldmacher and Kovtun, 2011). Such conjugates have received significant attention since positive results in breast cancer were reported for TDM-1 (Kadcyla; Roche), a conjugate of the monoclonal antibody trastuzumab and the cytotoxin maytansine (Verma et al., 2012; Dhillon, 2014). SGN-75, a humanized anti-CD70 monoclonal antibody conjugated to monomethyl auristatin F (MMAF) through a maleimidocaproyl (mc) linker (mcMMAF), has been evaluated in a phase 1 clinical trial for mRCC and for relapsed or refractory non-Hodgkin lymphoma (NCT01015911)^4^. Encouraging antitumor activity has been observed in both mRCC patients and in relapsed/refractory non-Hodgkin lymphoma patients.

**FIGURE 3:**
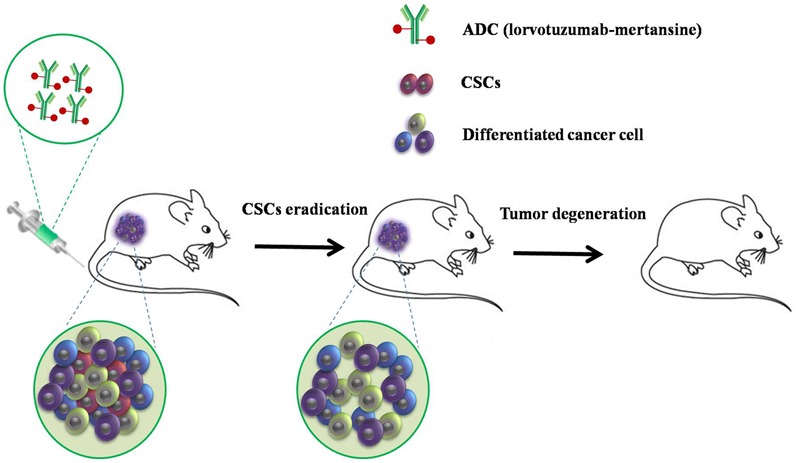
**A strategy to target human NCAM^+^ cells with lorvotuzumab mertansine.** Targeted therapy resulted in loss of Wilms’ tumor CSCs and a reduction in tumor size in the majority of cases, both *in vitro* and *in vivo*, followed by complete tumor degradation.

Wilms’ tumor (WT), a common pediatric solid renal tumor, is formed when renal precursor cells fail to differentiate properly. Pode-Shakked et al. (2013) successfully isolated and characterized WT CSCs expressing NCAM from human WT xenografts, and showed that lorvotuzumab mertansine (mertansine, also known as DM1, is a derivative of maytansine), an anti-NCAM antibody/cytotoxic drug conjugate, can dramatically reduce the expression of NCAM in WT cells from 42 to 5%, in addition to inducing a significant reduction in the colony forming capacity of treated cells (Pode-Shakked et al., 2013). A follow-up study showed that targeting the human NCAM^+^ cell fraction with lorvotuzumab-mertansine resulted in the loss of WT CSCs and induced a reduction in tumor size in the majority of cases, both *in vitro* and *in vivo*, followed by complete tumor degradation (**Figure 3**) (Pode-Shakked et al., 2013; Shukrun et al., 2014). A phase 2 trial evaluating lorvotuzumab-mertansine is ongoing (NCT02452554). Therefore, it can be concluded that using ADCs to deplete the stemness properties of CSCs by targeting ligands on the CSC cell surface may improve the impact of conventional chemotherapy. This would consequently result in a clinically relevant benefit for patients.

## Nanomedical Strategies

Recently, molecularly directed nanomedicine has become an appealing option as an RCC targeted therapy. Nanomedical strategies can control drug delivery and release more efficiently than other methods. Nanoparticles are high-capacity carriers for active compounds. They can easily accumulate at tumor sites due to the high porosity of the tumor vasculature, impaired lymphatic drainage (known as the enhanced permeability and retention effect), or the high affinity of the nanoparticle to markers specifically expressed on cancer cells. The typical nanomedicine delivery vehicles include liposomes, polymeric nanoparticles, nucleic acid based nanoparticles, dendrimers, magnetic nanoparticles, nanoshells, and virus nanoparticles.

## Liposomes

Liu et al. (2015a,b) systemically evaluated the *in vitro* performance of sorafenib-loaded poly (lactic-co-glycolic acid) (PLGA) nanoparticles, 1, 2-dipalmitoyl-sn-glycero-3-phosphocholine (DPPC) liposomes, and hydrophobically modified chitosan (HMC)-coated DPPC as delivery systems in the treatment of RCC. The results of this study showed that sorafenib-loaded PLGA nanoparticles and HMC-coated DPPC liposomes could kill significantly more tumor cells than sorafenib alone at lower concentrations; these methods could be further developed as unique clinical modalities and paired with ablative particles for synergistic treatments (Liu et al., 2015a,b). Recently, Kulkarni et al. (2016) showed that liposomes encapsulating a multi-receptor tyrosine kinase inhibitor (XL184) significantly increased anti-tumor activity than XL184 alone. They also found that the mechanism of XL184 liposomes’ anti-tumor activity is through inhibition of phosphorylation of Met, AKT, and MAPK pathways in RCC cells (Kulkarni et al., 2016). These results indicated that use of liposomes for multi-kinase pathways inhibition may emerge as a promising treatment option for RCC.

In another study, Kibria et al. (2016) addressed the discrepancy of antitumor effect of PEGylated liposomal Doxorubicin (PEG-LP (DOX)) in two tumor models. PEG-LP (DOX) failed to suppress the growth of the RCC OSRC-2 xenograft tumors. However, it could suppress breast cancer MDA-MB-231 xenograft tumor growth. Furthermore, they showed that extracellular matrix (ECM) molecules of MDA-MB-231 tumor limited the extent of the penetration and distribution of PEG-LP, which in turn enhancing the delivery of DOX to tumor vasculature. Therefore, these findings highlight the unique characteristics of tumor microenvironment that could be exploited by nanomedicine for developing novel drug delivery systems (Kibria et al., 2016).

## Polymeric Nanoparticles

Sumitomo et al. (2008) have used polymeric micelles incorporated with 7-Ethyl-10-hydroxy-camptothecin (SN38) to treat the RCC xenograft murine model. This nanoparticle significantly reduced the bulk of tumors and decreased the metastatic nodule number than free drug alone (Sumitomo et al., 2008). New studies showed that CRLX101, a nanoparticle-drug conjugate containing camptothecin conjugated to a biocompatible cyclodextrin/polyethylene glycol (PEG) copolymer, is currently being evaluated pre-clinically and clinically in multiple tumor types (Gaur et al., 2014; Lin et al., 2016). A phase I trial of CRLX101 in combination with bevacizumab for mRCC therapy has recently been completed (NCT01625936^5^). The preliminary data showed that CRLX101 combined with bevacizumab is a safe treatment in mRCC. This combination fulfilled the protocol’s predefined threshold for further examination with responses and prolonged PFS in a heavily pretreated population (Keefe et al., 2016). A randomized phase II clinical trial in mRCC of this combination is ongoing (NCT02187302).

Several studies have proposed to target RCC using nanocarriers, but, to date, these approaches have not been investigated in renal CSCs. Nanoparticles effectively inhibit multiple types of CSCs by targeting specific markers, including ALDH, CD44, and CD133, and/or specific signaling pathways and other key developmental signaling pathways implicated in the maintenance of the CSC pool of many tumors (Lu et al., 2016). Nanomedical strategies for renal CSCs are under research and development and a comprehensive evaluation of the approaches should be available soon.

As mentioned above, renal tumors from patients show strong resistance to conventional anti-cancer drugs such as interferon α and interleukins. TKIs have significantly improved outcome in patients with metastatic disease, while drug resistance still prevails in most patients over time because of the intricate tumor evasion mechanisms. TRC105 and CXCR4 inhibitors seem to be the promising drugs for renal tumors underlining the importance of specific surface markers (CD105 and CXCR4) of renal CSCs. According to the newly discovery of specific expression pattern in renal CSCs, some specific markers are further developed for obtaining the benefits of less resistance and enhanced therapy. And great interests are emerging to develop new therapeutic drugs or immunotoxins targeting renal CSCs based on molecular mechanisms that regulate stem cell properties. Despite progress being made in developing targeted strategies for renal cancer and renal CSCs, more approaches are still need to be explored notwithstanding the numerous impediments.

## Conclusion

Increasing evidence has shown that CSCs play a key role in cancer drug resistance, radiation therapy resistance, and are responsible for disease recurrence. With the development of the CSC hypothesis, new concepts and approaches for eradicating malignancies have emerged. It has been shown that renal carcinomas possess CSCs with mesenchymal properties, and self-renewal and multi-differentiation properties, although a definitive selection marker for their isolation and targeting remains to be found. Subpopulations of renal CSCs may coexist within a heterogeneous tumor, which means that renal CSC targeting therapy is a complicated science. A vast body of targeted therapies has been developed, and the systemic treatment of RCC has advanced in recent years. These advances have focused on inhibition of the VEGF and mTOR pathways, markers (such as CD105 and CXCR4), and differentiation of renal CSCs. During the last few years, numerous new clinical trials have been undertaken, or are currently recruiting. Even if obvious limitations exist in reaching renal CSCs *in vivo* owing to the inaccessibility of the entire tumor area or because of the tumor microenvironment, these limitations may be overcome with the use of ADCs and nanomedical strategies. Multimodal CSC-targeting strategies combined with new pathway specific modulators and cytotoxic agents may also contribute to overcoming the limitations in RCC treatment. Successful therapeutic exploitation of renal CSCs will help us to better understand the role of CSCs in different treatment responses, different stages of disease, and different patients. Precise identification of renal CSCs populations and the complete cell hierarchy will enable the accurate characterization of disease subtypes. This will ultimately lead to more personalized and targeted therapies. Rationally designed targeting therapy for RCC unseals new directions for exploration and clinical investigation and plants the seeds of hope for advanced clinical care.

## Author Contributions

Z-XY summarized the literature and wrote the manuscript. JM provided critical comments and wrote the manuscript. GZ and GS revised the manuscript and drew figures. H-LF and WZ supervised all the works and provided critical comments.

## Conflict of Interest Statement

The authors declare that the research was conducted in the absence of any commercial or financial relationships that could be construed as a potential conflict of interest.

## Footnotes

1 https://clinicaltrials.gov/

2 https://clinicaltrials.gov/

3 https://clinicaltrials.gov/

4 https://clinicaltrials.gov/

5 https://clinicaltrials.gov/
